# Full-Arch Rehabilitation Using Implant-Guided Surgery and Ozone Therapy to Prevent Complications in a Systemically Compromised Patient

**DOI:** 10.7759/cureus.76251

**Published:** 2024-12-23

**Authors:** Márcio de Carvalho Formiga, Iuri Martins, Amanda Randazzo, Cesar Guimarães, Leonardo Faverani

**Affiliations:** 1 Oral Implantology, Univali São José, Florianopolis, BRA; 2 Oral Diagnosis, UNICAMP (Universidade Estadual de Campinas), Piracicaba, BRA

**Keywords:** full-arch rehabilitation, guided implant surgery, implant osseointegration, ozone therapy, systemic inflammatory disease

## Abstract

Computer-guided surgery is a new technology in the field of implant dentistry. The surgical guide is produced using cone-beam computed tomography along with the patient’s intraoral scanning, with both documents integrated into software to produce the guide. It is important to note that surgery guided by tomography aims to achieve better diagnosis, planning, surgical precision, and prognosis. Additionally, it provides the dentist with greater predictability in rehabilitation with dental prostheses. Furthermore, less invasive procedures have become a trend in implant dentistry, and flapless guided surgery is a valuable tool in less traumatic surgical approaches. Rehabilitation with dental implants, regardless of the technique used, is closely interconnected with the osseointegration process. For this process to occur, all cells responsible for the bone-remodeling mechanism must function properly. However, individuals using drugs such as bisphosphonates experience impaired bone remodeling, necessitating careful clinical management of these patients. Given this context, the objective of our work is to report a case of guided dental implant surgery for the installation of an upper full-arch rehabilitation over implants in a patient with a history of bisphosphonate use and an indication for flapless surgery.

## Introduction

Guided surgery differs from static technology in that a surgical guide is produced using cone-beam computed tomography to guide the installation of implants or even osteotomies. It also differs from dynamic technology, where software transmits the patient’s images in real time through optical tracking [1]. Given this, it is important to highlight that tomography-guided surgery aims to achieve better diagnosis, planning, surgical precision, and improved prognosis. It also enables greater predictability in rehabilitation with dental prostheses [2].

Less invasive procedures have become a trend in implant dentistry. Flapless guided surgery has been documented, offering new perspectives with less aggressive approaches. Among its advantages are lower morbidity for the patient due to the absence of periosteum detachment, increased comfort in the immediate post-surgical period, surgical and prosthetic predictability, reduced surgical procedure time, and overall improved patient experience [3].

Success in rehabilitation with dental implants, regardless of the technique used, is closely interconnected with the osseointegration process. For this to occur, all cells responsible for the bone-remodeling mechanism must function properly. Additionally, the formation of immature bone depends on the adequate development of new blood vessels to supply the nutrients necessary for bone formation [4]. Patients undergoing treatment for bone pathologies or neoplasms who have used or are using antiresorptive drugs, such as bisphosphonates and denosumab, experience impaired osteoclast differentiation, leading to osteoclast apoptosis. Consequently, this results in decreased bone remodeling and resorption. Similarly, patients using antiangiogenic drugs face impaired blood vessel neoformation [5].

These patients are at risk of developing bone necrosis (BN), often occurring after dental interventions [4]. For this reason, clinical management and surgical interventions in such patients must be carefully analyzed, considering clinical consequences such as poor healing after extraction, difficulty managing bone tissue, pain, soft tissue edema, paresthesia, and exposed bone-all of which are challenging to resolve and control [4,6]. The oral environment is particularly vulnerable to BN due to the high demands of bone remodeling and elevated infection rates [7]. BN is a significant complication, especially in patients exposed to head and neck radiotherapy, corticosteroids, and antiresorptive and antiangiogenic agents, with its morbidity closely linked to systemic comorbidities.

Ozone therapy has emerged as an alternative treatment demonstrating improvements in the clinical condition of patients affected by BN. Studies have shown its effectiveness in enhancing quality of life and clinical outcomes [8]. Ozone has properties that contribute to bone regeneration, such as its disinfectant and anti-inflammatory effects, activation of intracellular metabolism in the oral mucosa, and promotion of regional angiogenesis. It stimulates the humoral immune system, favoring the synthesis of biologically active substances, such as interleukins, leukotrienes, and prostaglandins, thereby reducing inflammation and supporting cell regeneration. Ozone also increases phosphorus dioxide (PO_2_) levels in tissues and enhances local oxygenation [9].

In this study, we report a case of guided dental implant surgery in preparation for the subsequent installation of a superior protocol prosthesis in an immunocompromised patient who was treated with ozone therapy to avoid BN.

## Case presentation

**Figure 1:**
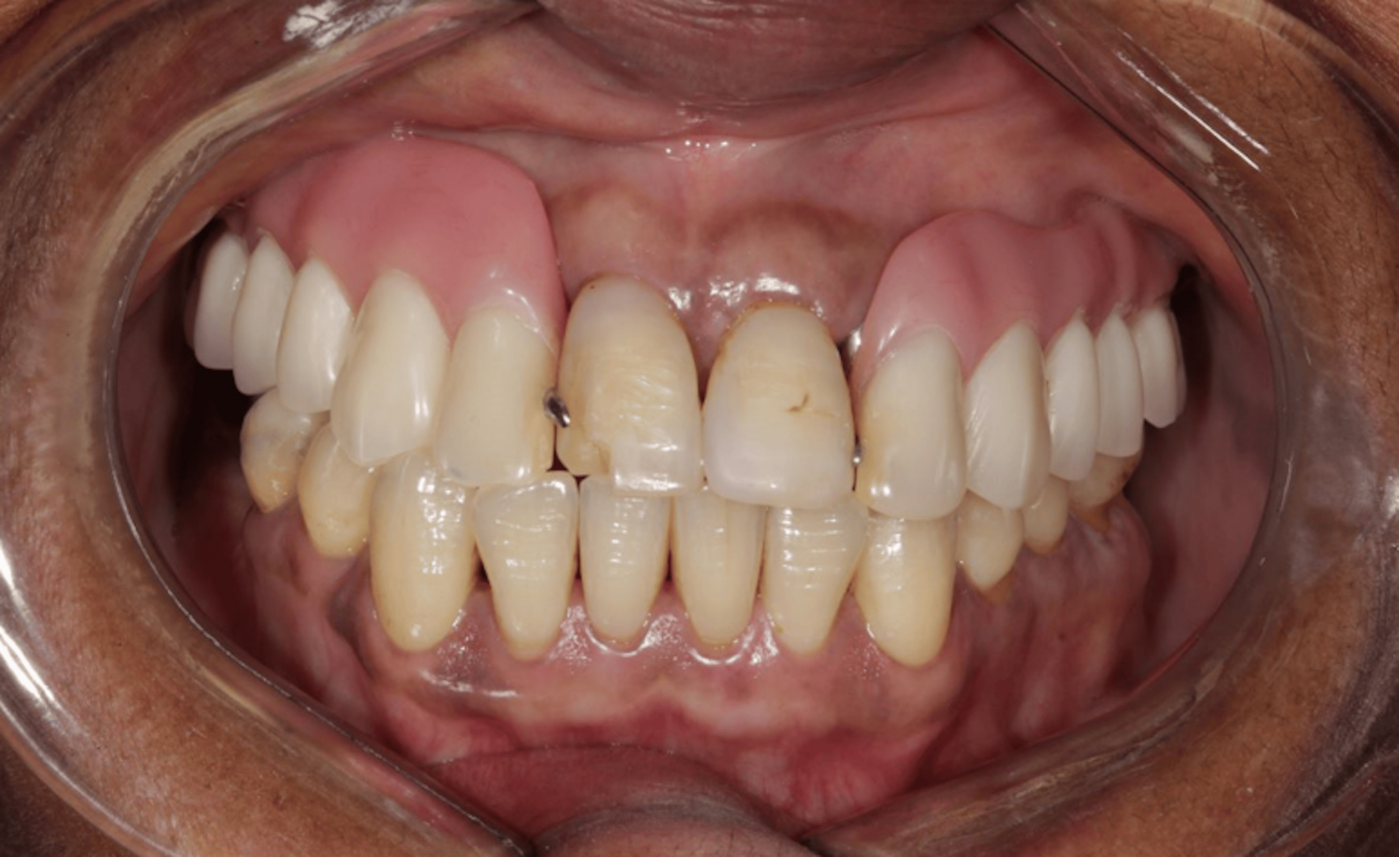
Buccal view of the patient’s current rehabilitation

**Figure 2:**
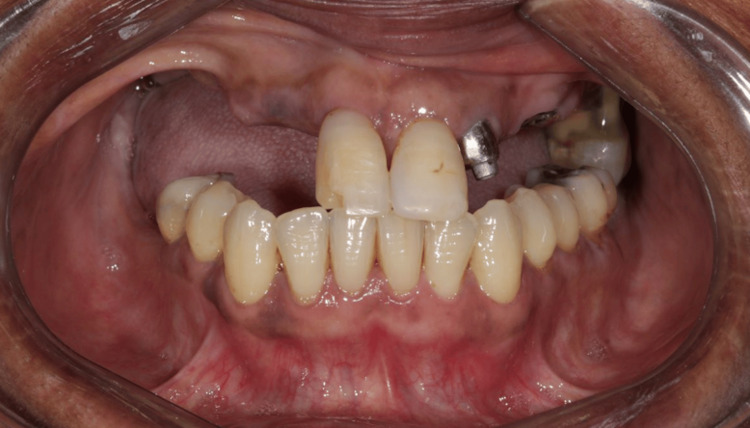
Buccal view of the patient’s condition without the removable prosthesis

A 60-year-old female sought care at the University Clinic of Implant Dentistry at UNIVALI (Universidade do Vale do Itajaí) in March 2023. During a thorough anamnesis, the patient reported using sodium alendronate 70 mg, one tablet per week, for over 20 years due to skeletal dysfunctions and rheumatoid arthritis. She also mentioned taking methotrexate 2.5 mg, amiodarone 200 mg, atorvastatin 20 mg, and acetylsalicylic acid 100 mg. No history of smoking or tobacco use was reported. The patient required maxillary rehabilitation with a prosthetic protocol because she had an upper removable prosthesis supported by the two central incisors and the left second molar, all of which showed periodontal compromise and were indicated for extraction (Figures 1, 2).

Due to her systemic conditions, the patient was warned about the risk of BN and initiated on an ozone therapy protocol, in addition to a six-month “drug holiday” from alendronate sodium as recommended by her rheumatologist.

**Figure 3:**
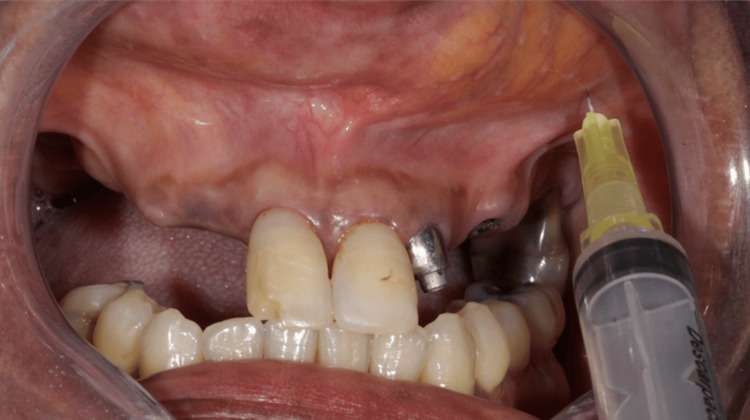
Ozone gas application

**Figure 4:**
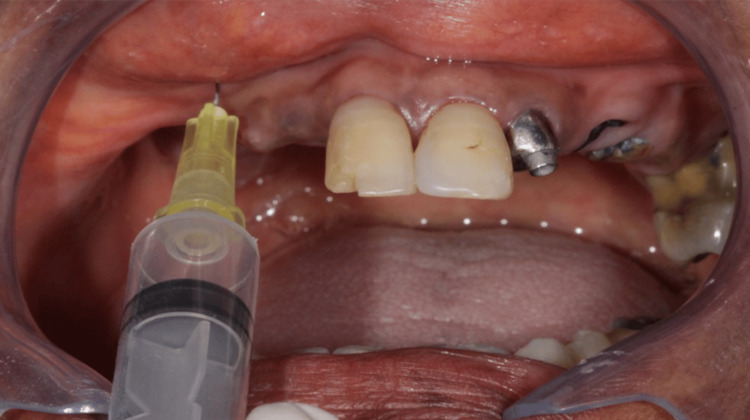
Ozone gas application

**Figure 5:**
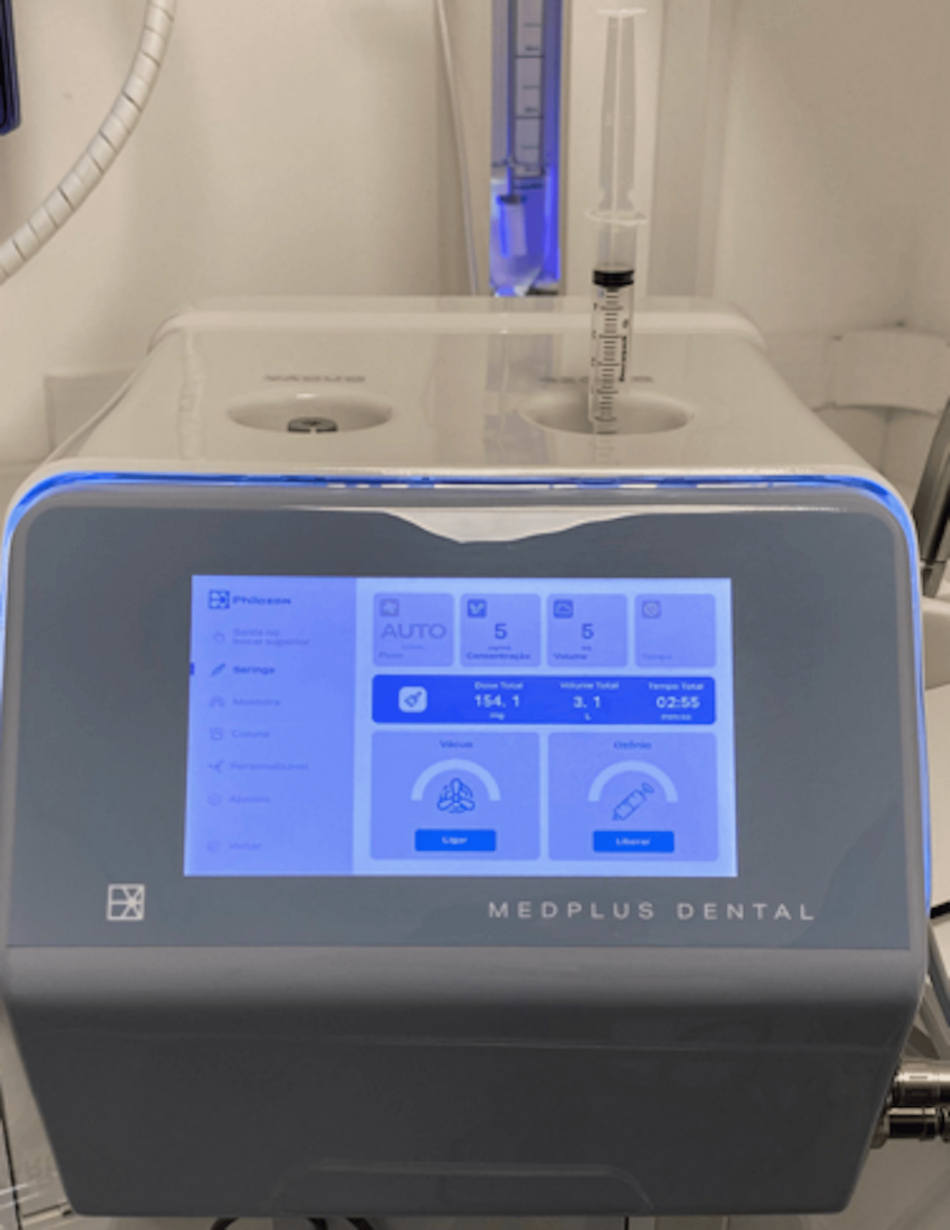
Ozone generator MedPlus (MedPlus Dental, Philozon-Balneário Camboriu, Brazil) producing ozone gas for administration

The patient underwent all stages of impressions. A surgical guide was created during the suspension of medication, and ozone therapy was performed on the maxillary region (Figures 3, 4). Weekly therapy sessions began and continued until the day of surgery. The ozone protocol consisted of gas applications (MedPlus Dental, Philozon-Balneário Camboriu/Brazil) of 5 mcg/mL, 1 mL per point, with one point per tooth region, on the buccal mucosa (Figure 5).

**Figure 6:**
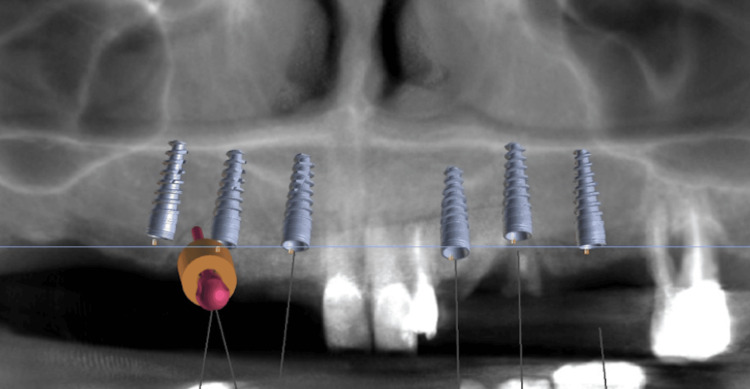
Implant positioning planning

**Figure 7:**
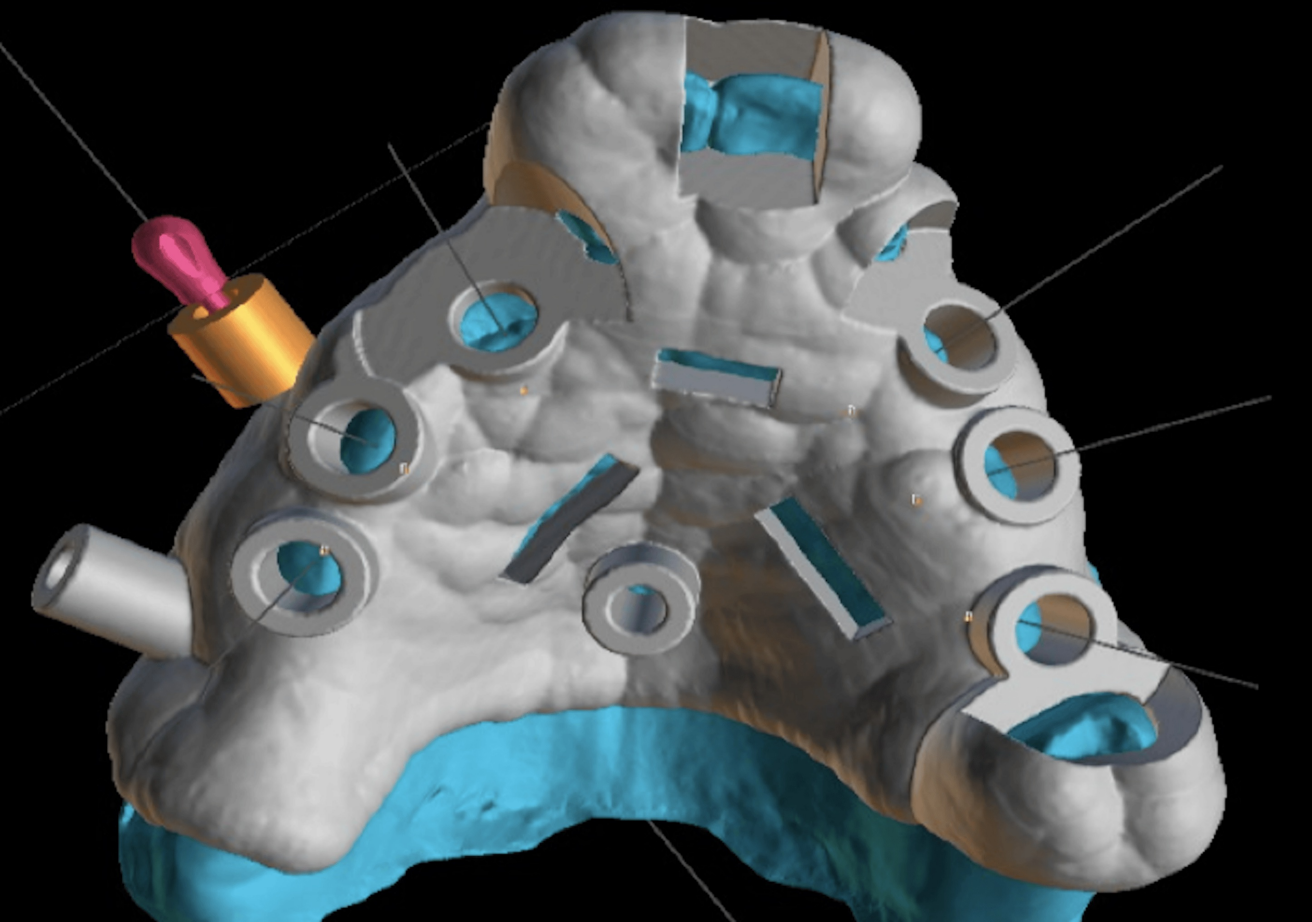
3D surgical guide planning

**Figure 8:**
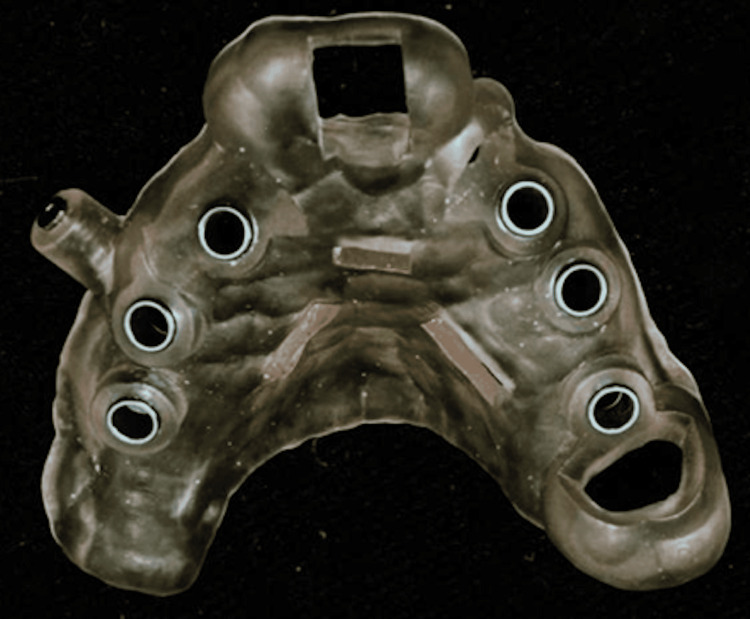
Printed surgical guide

The patient underwent a tomography scan and an intraoral scan to plan the 3D-printed surgical guide. Following these steps, the patient’s DICOM and STL files were uploaded into the Blue-Sky Bio program, where virtual implant placement was performed (Figure 6) and the virtual surgical guide was designed for 3D printing (Figures 7, 8). To minimize surgical and prosthetic trauma, a flapless surgery was planned.

**Figure 9:**
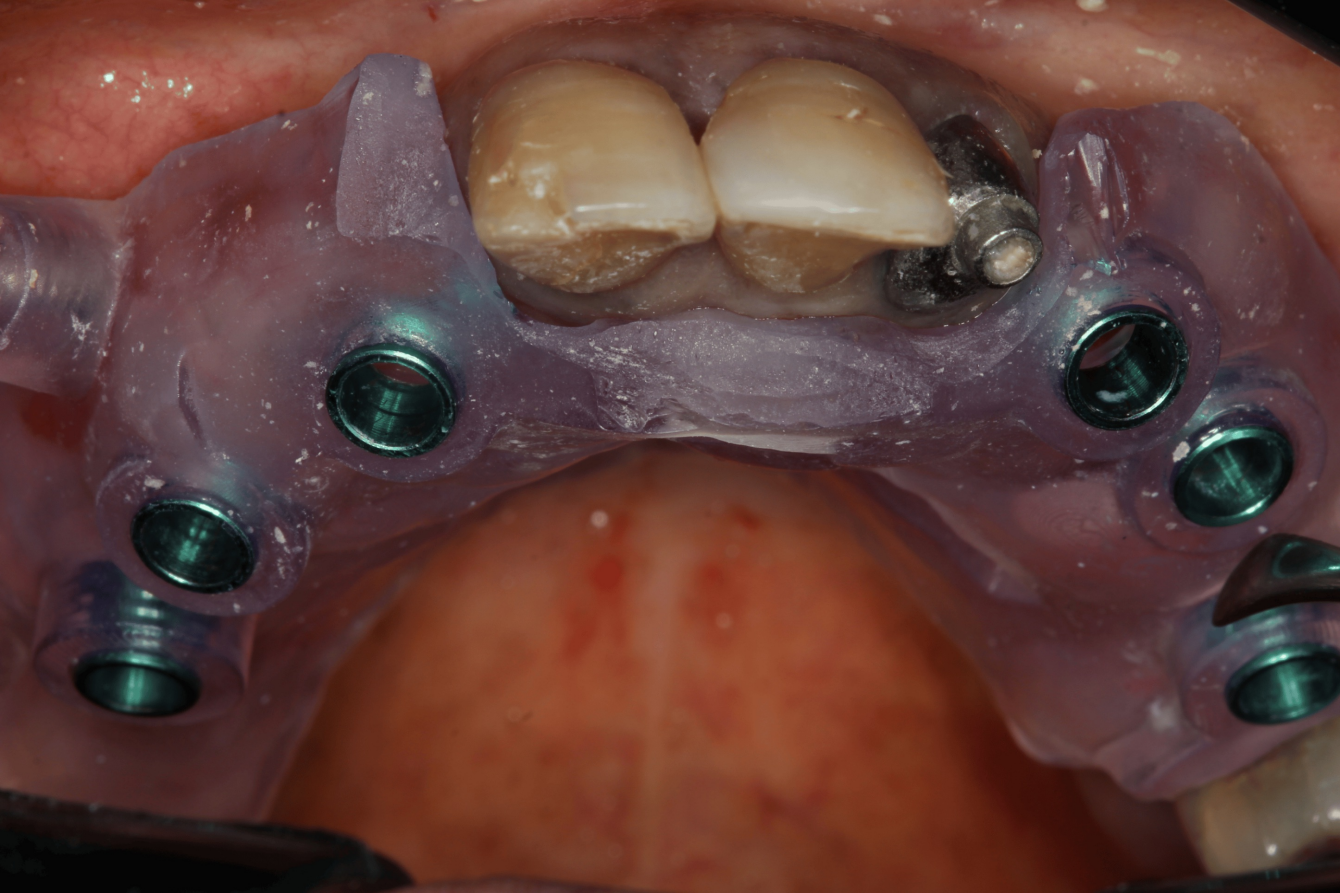
Surgical guide in position

**Figure 10:**
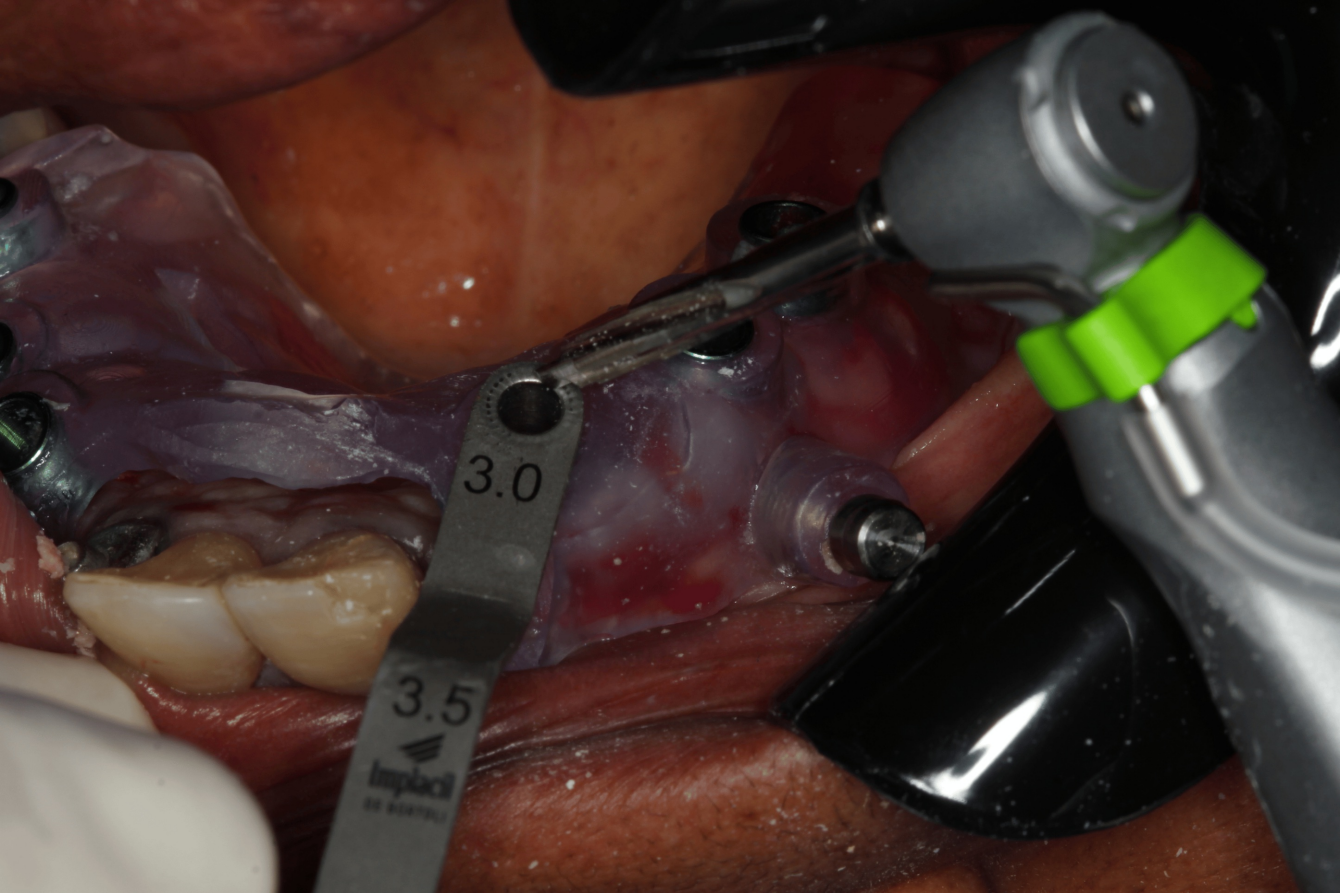
Transoperative view of the drilling through the surgical

**Figure 11:**
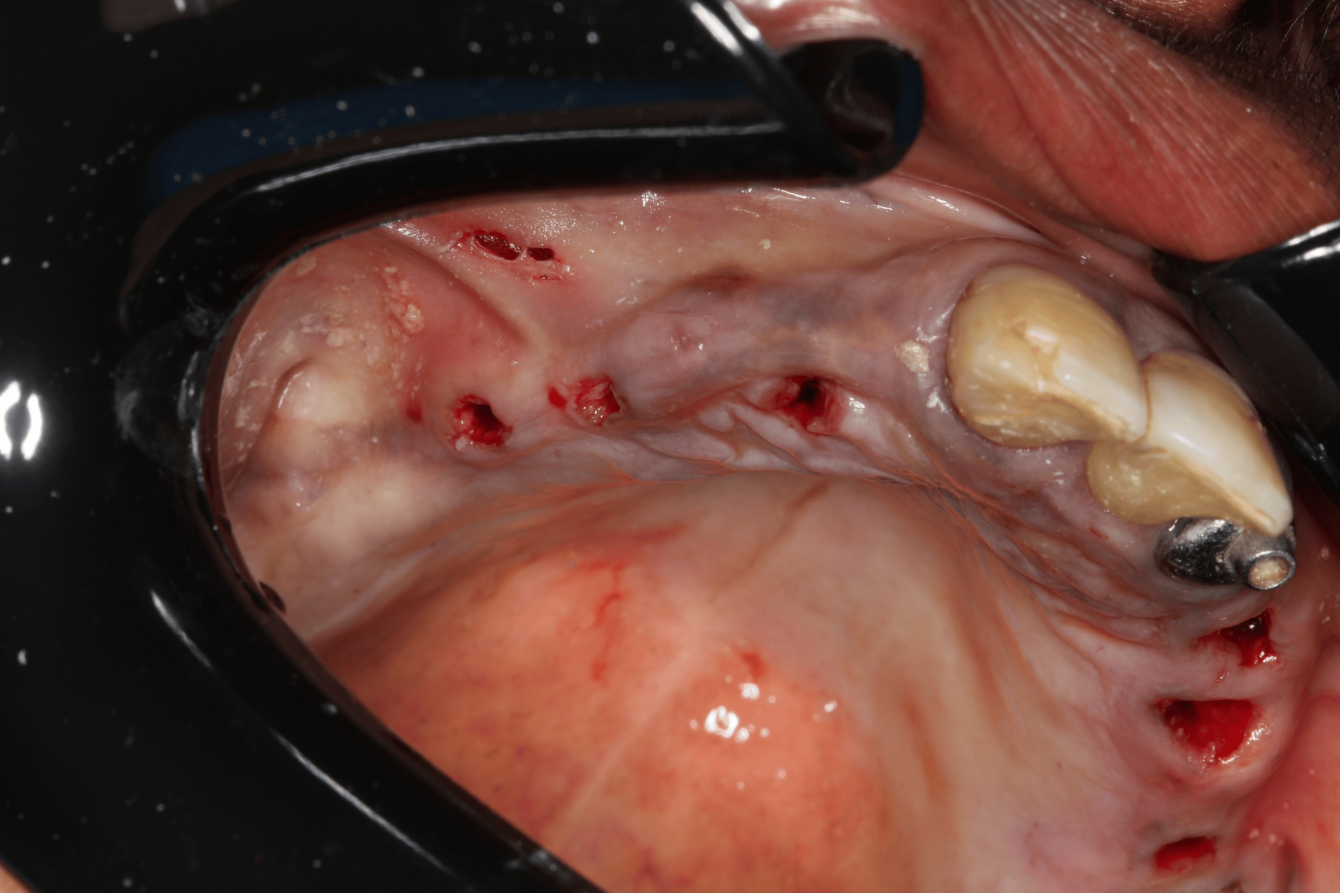
Immediate view of the mucosa after implant placement on the right side

**Figure 12:**
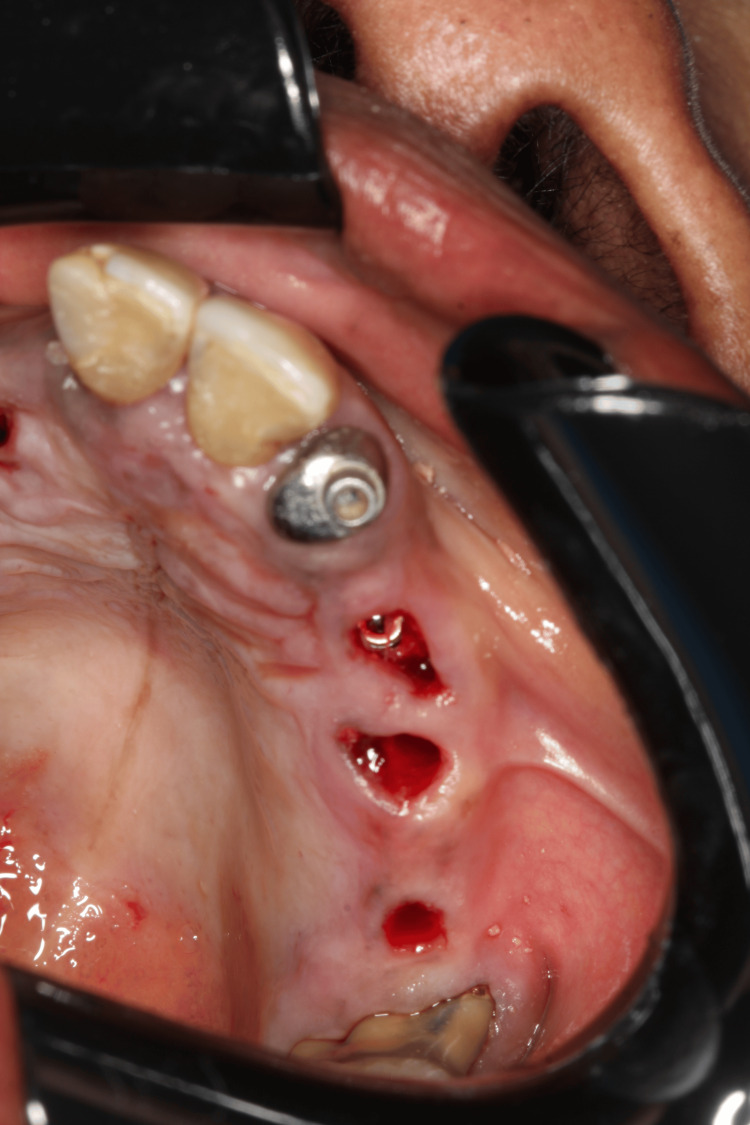
Immediate view of the mucosa after implant placement on the left side

**Figure 13:**
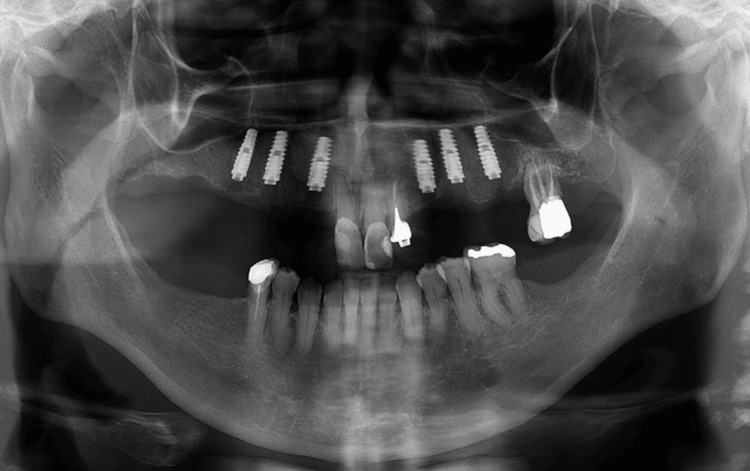
Panoramic radiograph seven days after implant placement

We planned the placement of six implants while maintaining the remaining teeth to support the removable prosthesis during the healing period. The surgery was performed four months after the initiation of ozone therapy. One hour before the procedure, we administered antibiotic prophylaxis with amoxicillin 500 mg, as suggested by the patient’s rheumatologist to prevent infection. Anesthesia was administered to block the bilateral posterior maxilla, bilateral infraorbital, palatine, and nasopalatine nerves, after which the surgical guide was fixed. After confirming proper adaptation of the surgical guide (Figure 9), drilling was performed for the placement of six Morse taper implants (Due Cone, Implacil de Bortoli-São Paulo, Brazil) (Figures 10-12). On the seventh day after surgery, the patient was recovering well, the soft tissue was healing appropriately, and a panoramic radiographic examination was performed (Figure 13).

**Figure 14:**
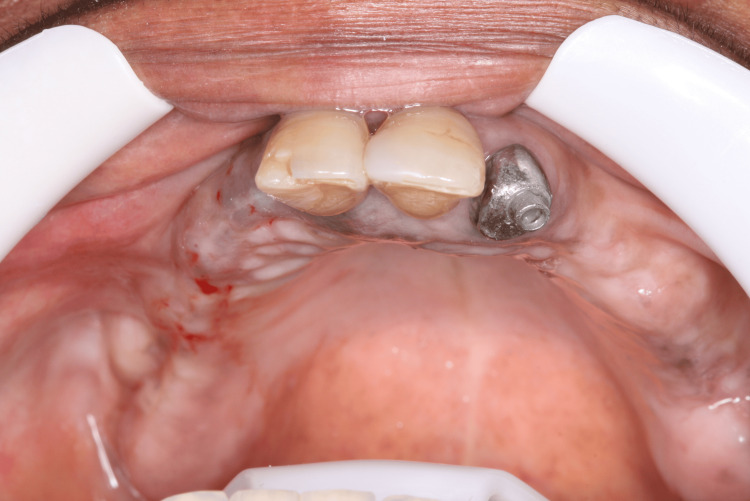
Four months after implant placement

**Figure 15:**
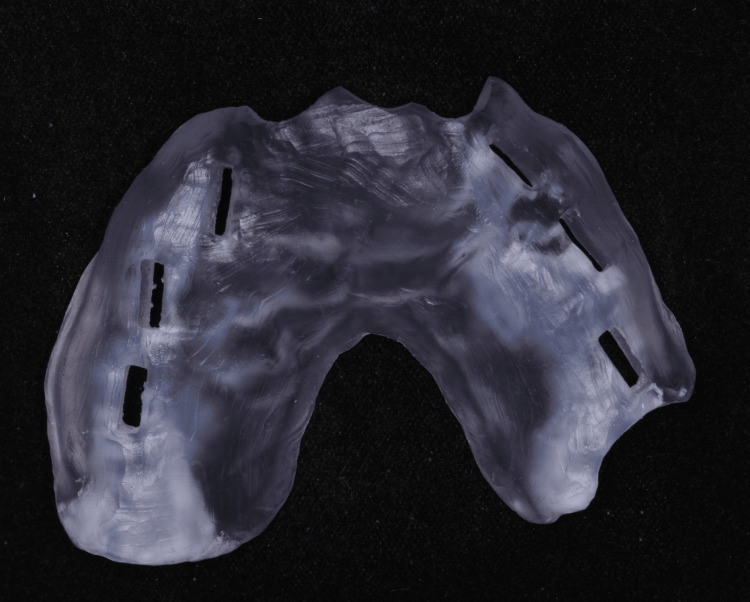
Second surgery printed guide

**Figure 16:**
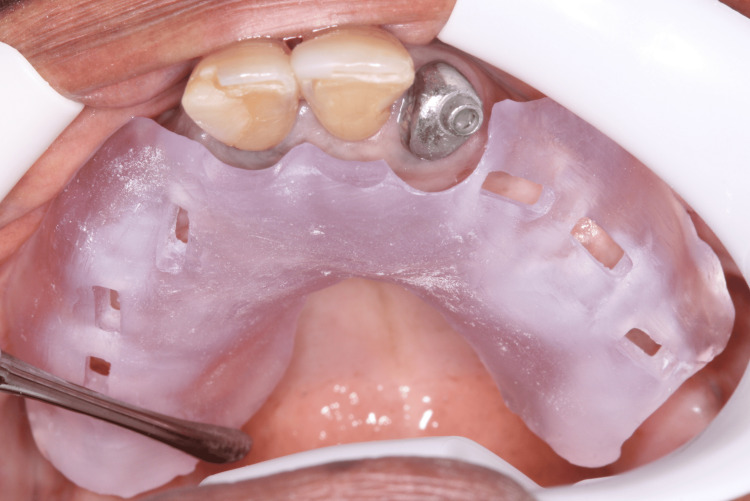
Second surgery printed guide in position

**Figure 17:**
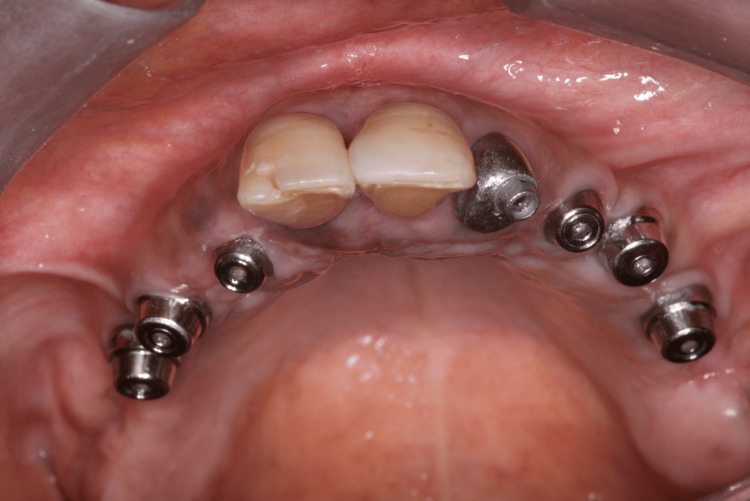
Abutments with healing caps installed

**Figure 18:**
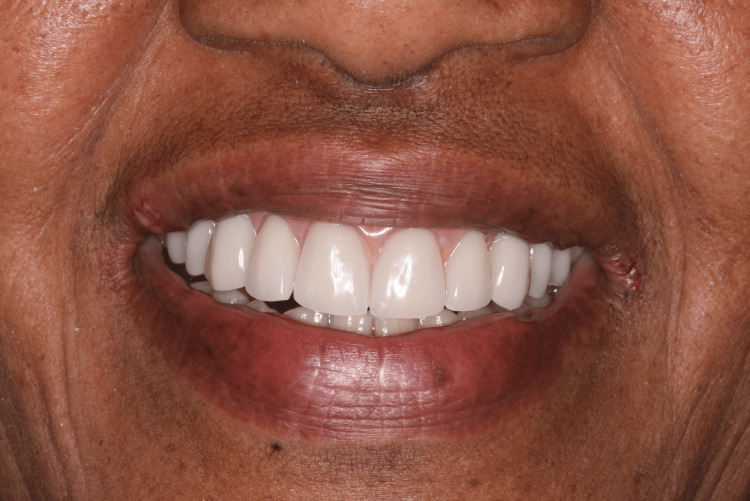
Extra-oral view of the final fixed rehabilitation over implants

**Figure 19:**
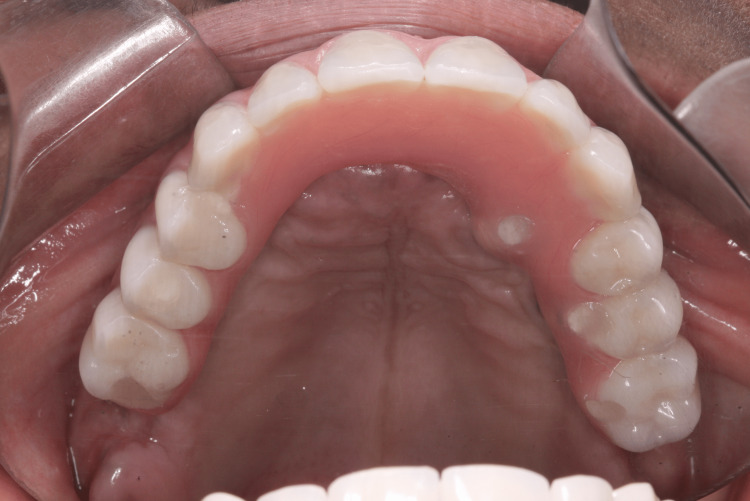
Occlusal view of the fixed screwed full arch rehabilitation over six implants

**Figure 20:**
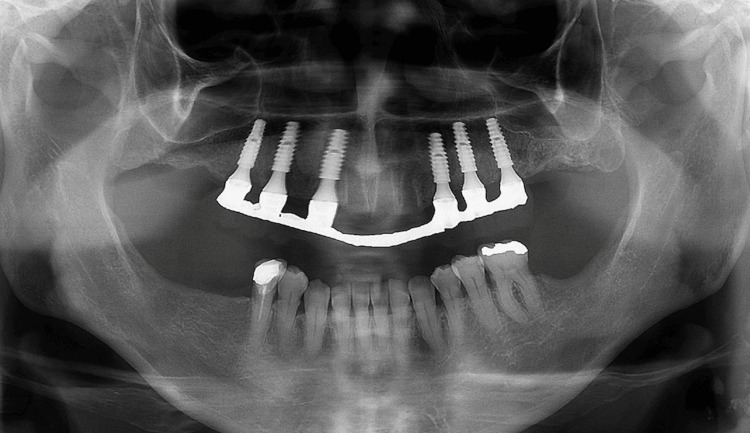
Twelve-month panoramic radiograph with the final prosthesis installed

Four months after implant placement, the patient returned for the reopening surgery (Figure 14). The patient continued with the ozone therapy protocol throughout the healing period. A new guide was printed for the second surgery, guided by the position of the implants from the first surgery, to avoid mucoperiosteal surgery and bone exposure (Figures 15, 16). Abutments with healing caps were installed on the six implants (Figure 17), and the remaining teeth were extracted. After 21 days, an impression of the implants was made to initiate prosthetic rehabilitation. Fourteen days later, the treatment was completed by screwing the full-arch fixed rehabilitation onto the implants (Figures 18, 19), with no apparent complications related to the medication. A panoramic radiograph was performed six months later for follow-up (Figure 20).

## Discussion

Dental implants are widely used to replace lost teeth, with evidence demonstrating improvements in patients’ quality of life. When surgical procedures are performed using guided surgery, patients often perceive it as a novel technique, leading to heightened expectations. It is essential to communicate all relevant information about the guided surgery technique to the patient in an accessible manner, including its advantages and disadvantages. Among the most cited advantages are reduced edema and postoperative pain compared to the conventional technique, as well as the high precision of implant placement, which contributes to greater comfort and longevity of the treatment [6]. For these reasons, we chose guided flapless surgery in this case, avoiding mucosal and periosteal detachment, ensuring a less traumatic postoperative experience for the patient, and preserving tissue integrity.

A review [1] analyzed clinical trials from the past 10 years that investigated dental implant procedures performed using static or dynamic guided surgery, or a combination of both, and compared them to conventional implant placement, commonly referred to as “freehand.” The comparison parameters included implant stability, guide fracture, guide instability, bone dehiscence, postoperative outcomes, and osseointegration. After a specific statistical analysis, the authors concluded that differences existed between techniques regarding intraoperative complications and long-term post-surgical healing and osseointegration of dental implants in the short and long term [1].

In our case, we opted for guided surgery due to the systemic condition of the patient, who had rheumatoid arthritis and was undergoing treatment with weekly doses of alendronate sodium 70 mg. This approach allowed us to preserve tissues and install the implants in the least traumatic way possible.

A systematic review [2] analyzed 30 articles involving a total of 1,086 patients and 4,900 implants and did not find significant differences regarding these clinical issues. However, it is important to highlight the advantages of guided surgery in patients with limited bone tissue and favorable clinical evidence concerning pain, hemorrhage, and edema immediately after surgery [2].

Guided surgery is highly efficient in implant placement and in ensuring treatment predictability, as shown in a study comparing implant deviations in guided surgery [3]. That study involved 10 patients with edentulous maxillae and eight patients with edentulous mandibles. All participants underwent reverse planning, surgical guide creation, implant installation surgery, and the installation of a final prosthesis protocol [3]. In our case, we followed a similar approach for rehabilitation with an upper maxillary protocol prosthesis. This method caused minimal tissue damage and provided favorable prosthetic and surgical predictability for osseointegration of the implants, installation of the protocol prosthesis, and long-term treatment success. Following the surgery, a new tomography was performed and compared with the guide planning. After statistical analysis, no deviation was found in the implant placements. However, there was a greater tendency for deviation in the apical region of the implant and when placed in posterior regions. This demonstrates that guided surgery offers good predictability and is an effective tool that indirectly enhances the success of rehabilitation with dental implants and prosthetics [3].

In another study [10], the authors analyzed the deviation of implants placed through guided surgery. In that study, 109 implants were placed in 60 patients, and in all cases, prior tomography and surgical guidance were performed. In every procedure, no structures were damaged, and no complications occurred. Additionally, osseointegration was observed in all cases, and prostheses were subsequently installed on the implants. The authors concluded that the precision and accuracy of implant placement using guided surgery were adequate and had an excellent prognosis, particularly in aesthetic cases, cases with limited bone availability, flapless surgery, and immediate loading [10].

It is widely recognized that the success of rehabilitation with dental implants is closely linked to their osseointegration into the patient’s bone. For this process of bone remodeling to occur, osteoclasts must function properly. Therefore, patients using antiresorptive drugs are at risk for procedures that require bone remodeling, such as dental implants, due to the potential risk of developing osteonecrosis resulting from impaired cellular function [4]. As a result, the patient in our case presented a potential risk of developing osteonecrosis due to her use of alendronate 70 mg for more than 20 years, combined with methotrexate, a potent immunosuppressor, for rheumatoid arthritis, monitored by a rheumatologist.

In a case report demonstrating a positive outcome with dental implants in a patient using bisphosphonates, the use of these medications was identified as a risk factor for osteonecrosis, particularly related to the mode and duration of drug use. Patients taking bisphosphonates must undergo rigorous anamnesis and a risk-benefit analysis for surgery, with protocols to minimize potential complications, such as medication discontinuation, antibiotic prophylaxis, and reduced surgical and prosthetic trauma [11]. Consequently, the patient in our case was advised by her rheumatologist to discontinue the medication for more than six months. Additionally, she underwent prophylactic ozone therapy, administered locally through injections and via inhalation. Despite these precautions, flapless guided surgery was chosen to minimize surgical and prosthetic trauma.

The authors [11] reported a case involving implant and prosthesis placement in the region of teeth 11 and 21 for a patient using ibandronate and risedronate to treat osteopenia. The patient discontinued these medications under the guidance of an orthopedist for three months before surgery and three months afterward, totaling six months without medication [11]. A recent clinical study [12] demonstrated that ozone treatment could positively impact post-extraction sites, particularly in patients at risk of developing Medication-Related Osteonecrosis of the Jaw (MRONJ), likely due to its effects on improving tissue oxygenation, supporting wound healing, stimulating the immune system, and activating angiogenesis in inflamed tissue [13]. Ozone has also been associated with bone healing in some studies, owing to its immunomodulatory effects, which can reduce inflammation and pain [13].

Clinical evidence indicates that intravenous (IV) bisphosphonates pose a greater risk compared to oral administration [14]. According to the American Association of Oral and Maxillofacial Surgeons, dental implant surgery is contraindicated in patients undergoing IV bisphosphonate therapy. In our case, an important factor in choosing dental implant treatment was that the patient had been on oral bisphosphonate therapy administered once a week rather than IV therapy [14]. Our literature review, which evaluated 17,237 patients undergoing bisphosphonate therapy across 10 studies, revealed a total of 30,070 dental implants placed. Among these, only two studies reported cases of necrosis, while failure in implant osseointegration was more commonly observed and identified as a consequence of antiresorptive drug use. However, eight studies reported successful implant placement, concluding that while antiresorptive therapy is a risk factor for osteonecrosis, further clinical studies are necessary to establish the safety of dental implant surgery in these patients [15].

## Conclusions

Guided surgery without a flap offers an alternative in complex cases that require precision in implant placement, as well as in cases that demand reduced surgical morbidity without a full flap or periosteal detachment. In this case, a careful evaluation was required due to the patient’s long-term use of alendronate sodium for rheumatoid arthritis. Consequently, we opted for guided surgery without a flap and incorporated ozone gas therapy as prophylaxis during and after the surgical procedure, along with a six-month medication break under the guidance of the rheumatologist. Cases like this must be approached with caution to prevent subsequent complications. However, these prophylactic measures and strategies to minimize surgical trauma, such as prophylactic ozone therapy and flapless surgery, are not yet well-documented. More randomized studies are needed to verify their effectiveness, particularly in patients with a history of antiresorptive drug use.
